# A Novel Microbial Consortia Catalysis Strategy for the Production of Hydroxytyrosol from Tyrosine

**DOI:** 10.3390/ijms24086944

**Published:** 2023-04-08

**Authors:** Pengfei Gong, Jiali Tang, Jiaying Wang, Chengtao Wang, Wei Chen

**Affiliations:** Key Laboratory of Geriatric Nutrition and Health, Ministry of Education, Beijing Advanced Innovation Center for Food Nutrition and Human Health, Beijing Engineering and Technology Research Center of Food Additives, School of Food and Health, Beijing Technology and Business University, Beijing 100048, China

**Keywords:** synthetic biology, HpaBC, enzyme promiscuity, microbial consortia

## Abstract

Hydroxytyrosol, a valuable plant-derived phenolic compound, is increasingly produced from microbial fermentation. However, the promiscuity of the key enzyme HpaBC, the two-component flavin-dependent monooxygenase from *Escherichia coli,* often leads to low yields. To address this limitation, we developed a novel strategy utilizing microbial consortia catalysis for hydroxytyrosol production. We designed a biosynthetic pathway using tyrosine as the substrate and selected enzymes and overexpressing glutamate dehydrogenase GdhA to realize the cofactor cycling by coupling reactions catalyzed by the transaminase and the reductase. Additionally, the biosynthetic pathway was divided into two parts and performed by separate *E. coli* strains. Furthermore, we optimized the inoculation time, strain ratio, and pH to maximize the hydroxytyrosol yield. Glycerol and ascorbic acid were added to the co-culture, resulting in a 92% increase in hydroxytyrosol yield. Using this approach, the production of 9.2 mM hydroxytyrosol was achieved from 10 mM tyrosine. This study presents a practical approach for the microbial production of hydroxytyrosol that can be promoted to produce other value-added compounds.

## 1. Introduction

The emergence of synthetic biology has accelerated and facilitated the construction of biosynthetic pathways and the heterogeneous production of value-added compounds in various hosts [1,2]. Microbial consortia have emerged as the next-generation strategy for the efficient production of biofuels and high-value natural metabolites such as paclitaxel precursors [3], *cis,cis*-muconic acid [4], stilbenes [5], and salidroside [6] in synthetic biology. Microbial consortia systems utilize at least two microorganisms to segregate processes for increased efficiency and perform complex functions that individual populations cannot [7]. They are more robust to fluctuations and interference and can overcome drawbacks of individual populations, including the ability to achieve a higher conversion of sugar mixtures and overcome the challenge of high-level intermediate production. Moreover, they can overcome the drawbacks of individual populations. For example, microbial consortia can achieve a higher conversion of sugar mixtures [8] and overcome the challenge of high-level intermediate production [9].

While previous research on microbial consortia systems has focused on the stability of mixed populations achieved by engineering either two cells of the same species or a synthetic microbial consortia [10], there are limitations to the biosynthesis of biochemical products in a microbial consortia system comprising only one microbial species. These limitations include an imbalance in the tradeoff of the enzymes in the pathway [11], yield of undesired byproducts due to the promiscuity of the pathway enzymes [12], and reduction in biosynthetic efficiency owing to the difficulty of optimizing different steps in the pathway [4]. Thus, the aim of this study is to design a “two-stage” microbial consortia catalysis strategy to overcome these challenges.

This study proposes a two-stage whole-cell catalysis of two *Escherichia coli* strains to accomplish the biosynthesis of hydroxytyrosol (3,4-dihydroxyphenylethanol) from tyrosine. Hydroxytyrosol is one of the most abundant phenolic compounds in olive fruits and virgin olive oil [13], has been proven to be a powerful antioxidant scavenger of free radicals, and confers cell protection [14], exhibiting antiatherogenic [15], anti-inflammatory [16], antimicrobial [17], and anticancer activities [18]. While chemical synthesis and enzymatic methods for obtaining hydroxytyrosol have been reported, they are limited by the high cost of reagents or enzymes [19]. Recently, microbial biotransformation of simple sugars, tyrosol, or tyrosine to hydroxytyrosol has been reported [20], and different artificial pathways have been established for hydroxytyrosol biosynthesis. The two-component flavin-dependent monooxygenase, HpaBC from *E. coli*, is the most widely used enzyme for hydroxytyrosol synthesis. However, the promiscuity of HpaBC usually causes the formation of L-dopa and loss of carbon sources, resulting in black cultures with low yields. While strategies such as the design of microbial co-cultures and removal of NH_4_Cl have been applied to alleviate this problem [21,22], the robustness of the co-culture system was difficult to maintain, resulting in a significant decrease in the cell density.

The two-stage microbial consortia catalysis strategy proposed in this study provides a good solution for limiting the promiscuous activities of the key enzymes in the biosynthetic pathway. This strategy facilitates the harness of the enzymes in the pathway and optimization of the segregated metabolic pathways, providing new perspectives in metabolic engineering and synthetic biology.

## 2. Results

### 2.1. Design of the Hydroxytyrosol Biosynthetic Pathway

**Figure 1:**
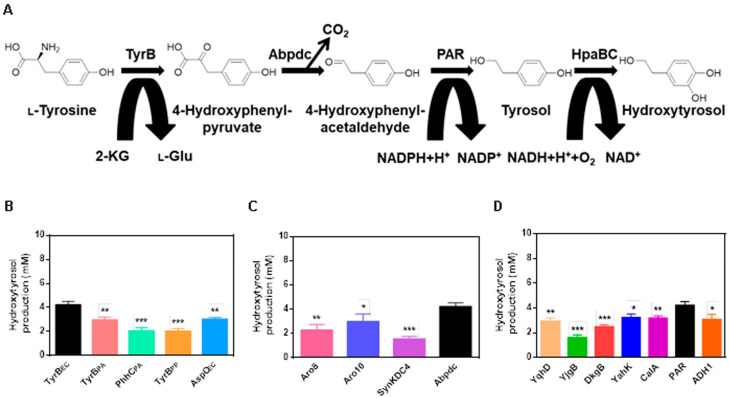
Production of hydroxytyrosol from tyrosine by whole-cell bioconversion. (**A**) Biosynthesis of hydroxytyrosol from tyrosine via the designed pathway. (**B**) Production of hydroxytyrosol by strains with different aminotransferases. (**C**) Production of hydroxytyrosol by strains with different 4-hydroxyphenylpyruvate decarboxylases. (**D**) Production of hydroxytyrosol by strains with different reductases. 10 mM tyrosine was supplemented as substrate. Data are expressed as the mean ± SD (*n* = 3). * *p* < 0.05, ** *p* < 0.01 and *** *p* < 0.001, compared to each control.

A heterologous hydroxytyrosol biosynthetic pathway in *E. coli* was designed, as illustrated in Figure 1A. The pathway involved the conversion of tyrosine to 4-hydroxyphenylpyruvate by the aminotransferase TyrB_EC_ from *E. coli* W3110, followed by oxidation to 4-hydroxyphenylacetaldehyde by the 4-hydroxyphenylpyruvate decarboxylase Abpdc from *Azospirillum* brasilense. The phenylacetaldehyde reductase Par from Rosa hybrid cultivar reduced 4-hydroxyphenylacetaldehyde to tyrosol, which was subsequently hydroxylated to produce hydroxytyrosol by the monooxygenase HpaBC from *E. coli* BL21 (DE3). To optimize the pathway, TyrB_EC_ was substituted by TyrB_PA_ or PhhC_PA_ from Pseudomonas aeruginosa, TyrB_PP_ from Pseudomonas putida, and AspC_EC_ from *E. coli* W3110, as shown in Figure 1B. The highest hydroxytyrosol yield was observed in the strain with TyrB_EC_. Furthermore, 4-hydroxyphenylpyruvate decarboxylase from Saccharomyces cerevisiae (Aro8 and Aro10), Komagataella phaffii GS115 (SynKDC4), and *A. brasilense* (Abpdc) were tested, with the strain expressing Abpdc displaying the highest hydroxytyrosol yield (Figure 1C). Finally, strains co-expressing TyrB_EC_ and Abpdc with various reductases, including YqhD, YjgB, DkgB, YahK from *E. coli* W3110, CalA from Pseudomonas nitroreducens, Par, and ADH1 from S. cerevisiae, produced varying levels of hydroxytyrosol, with a maximum yield of 4.21 mM observed in the strain expressing Par, as shown in Figure 1D.

### 2.2. Cofactor Optimization

**Figure 2:**
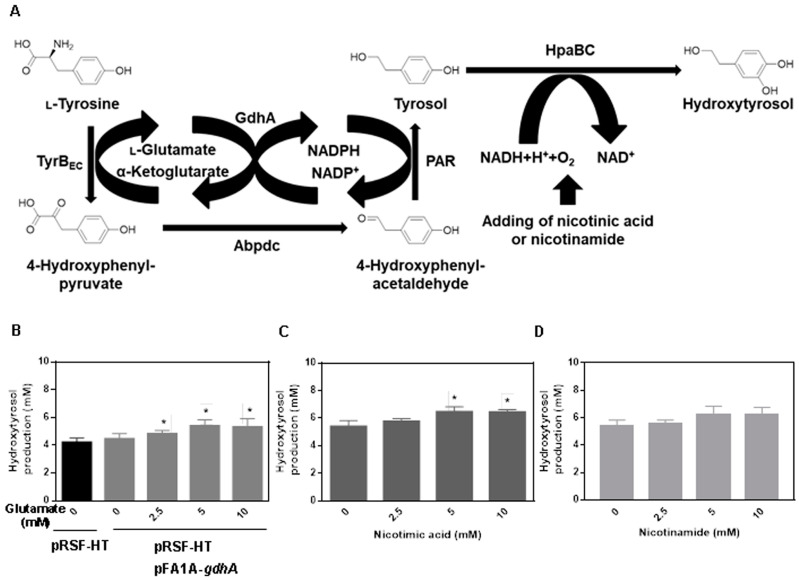
Optimization of cofactor involved in the production of hydroxytyrosol from tyrosine. (**A**) Scheme of hydroxytyrosol production by introducing GdhA and adding L-glutamate, nicotinic acid or nicotinamide to optimize cofactor supply. (**B**) Optimization of hydroxytyrosol production by introducing GdhA and addition of glutamate. (**C**) Optimization of hydroxytyrosol production by addition of nicotimic acid. (**D**) Optimization of hydroxytyrosol production by addition of nicotinamide. 10 mM tyrosine was supplemented as substrate. Data are expressed as the mean ± SD (*n* = 3). * *p* < 0.05, compared to the control.

To facilitate cofactor cycling and improve hydroxytyrosol biosynthesis, TyrB and Par were coupled by overexpressing glutamate dehydrogenase GdhA from *E. coli* W3110. This coupling was assumed to regenerate NADPH and recycle L-glutamate and α-ketoglutarate in the transamination step catalyzed by TyrB_EC_ (Figure 2A). As demonstrated in Figure 2B, the overexpression of GdhA increased the hydroxytyrosol yield, which was further enhanced by the addition of 2.5–10 mM L-glutamate. Additionally, as HpaBC is NADH-dependent, nicotinic acid or nicotinamide was introduced to the pathway to supplement the cofactor NADH to ensure sufficient reduction. The results shown in Figure 2C,D indicated that low concentrations of nicotinic acid and nicotinamide (5–10 mM) were effective in enhancing the production of hydroxytyrosol.

### 2.3. Design of a Microbial Consortia Catalysis Strategy

**Figure 3:**
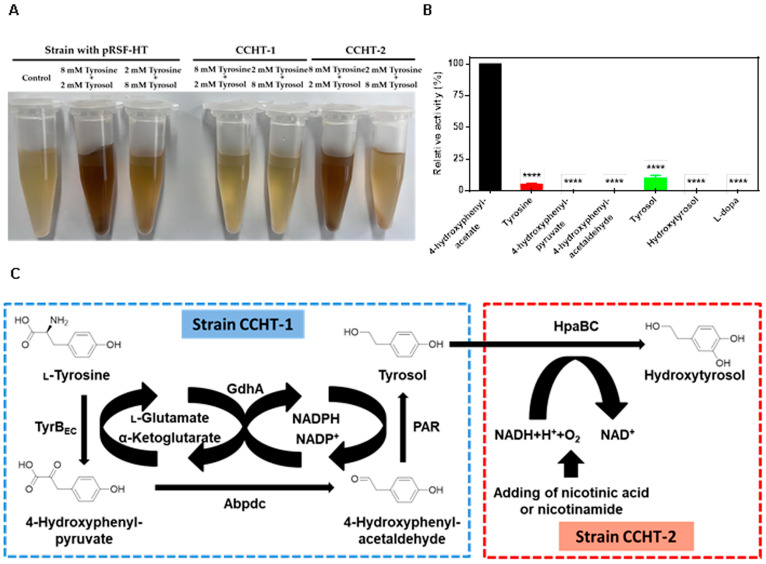
The promiscuity of HpaBC and design of a microbial consortia catalysis strategy. (**A**) The color change of strain with pRSF-HT plasmid, CCHT-1, and CCHT-2 cultured with total 10 mM substrate (tyrosin and tyrosol). (**B**) Catalytic activities of HpaBC towards different substrates. (**C**) Design of a microbial consortia catalysis strategy by dividing the biosynthetic pathway into two individual strains. Data are expressed as the mean ± SD (*n* = 3). **** *p* < 0.0001, compared to the control.

Due to the promiscuity of the monooxygenase HpaBC, the color of the culture broth turned dark brown during catalysis (Figure 3A). The protein HpaBC was expressed in a strain BL21(DE3) harboring plasmid pET28a-*hpaBC* (Figure S1), and its ability to catalyze both tyrosine and tyrosol was observed (Figure 3B). These findings underscore the promiscuous nature of HpaBC. To optimize the biosynthetic pathway, the original strain with plasmid pRSF-HT was divided into two separate strains with distinct functions in the biosynthetic pathway. The engineered strain with HpaBC demonstrated an evident color change in the culture broth at a high concentration of tyrosine when supplemented with different concentrations of tyrosine and tyrosol (Figure 3A). To further enhance hydroxytyrosol production, a microbial consortia catalysis strategy was proposed, where CCHT-1 and CCHT-2 strains were engineered to perform distinct parts of the biosynthetic pathway, with strain CCHT-1 converting tyrosine to tyrosol, and strain CCHT-2 catalyzing tyrosol to hydroxytyrosol (Figure 3C).

### 2.4. Fermentation Time Course of Two Individual Strains

**Figure 4:**
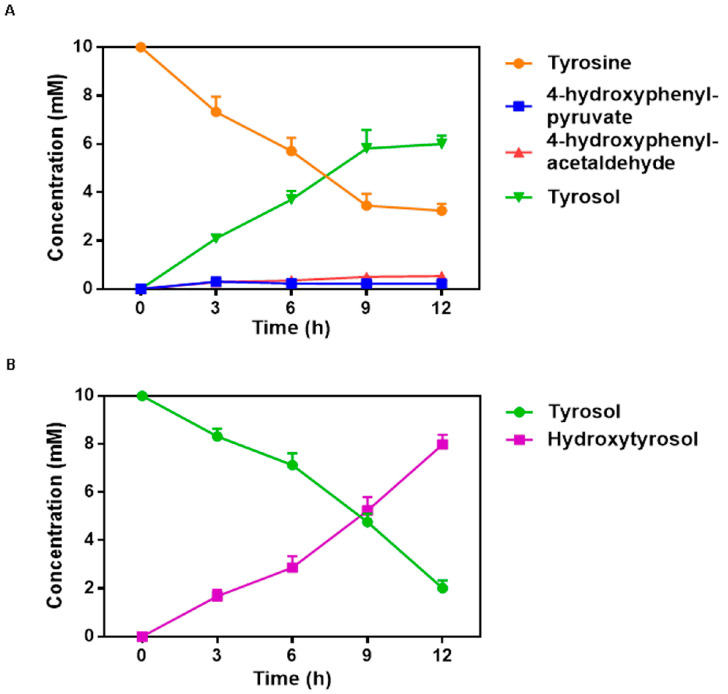
Fermentation time course of CCHT-1 and CCHT-2. (**A**) Time course of tyrosine, 4-hydroxyphenylpyruvate, 4-hydroxyphenylacetaldehyde and tyrosol during CCHT-1 fermentation. The orange closed circle, blue closed square, magenta closed triangle, and green closed triangle represents tyrosine, 4-hydroxyphenylpyruvate, 4-hydroxyphenylacetaldehyde, and tyrosol, respectively. (**B**) Time course of tyrosol and hydroxytyrosol during CCHT-2 fermentation. The green closed circle and purple closed square represent tyrosol and hydroxytyrosol, respectively. Data are expressed as the mean ± SD (*n* = 3).

As depicted in Figure 4A, the time course of tyrosol concentration, intermediates, and the consumption of the substrate tyrosine were analyzed in a culture broth contained the CCHT-1 strain bearing the plasmid pRSF-gdhA-tyrB_EC_-abpdc-par. The CCHT-1 strain was cultured in the presence of 10 mM tyrosine and following 12 h of fermentation, the tyrosol yield was 6.00 mM. In Figure 4B, the temporal changes in tyrosol and hydroxytyrosol concentrations were measured when the CCHT-2 strain expressing HpaBC was cultured. Upon supplementation with 10 mM tyrosol, the yield of hydroxytyrosol reached 7.99 mM after 12 h biotransformation. However, the culture broth rapidly darkened after 6 h of biotransformation when supplemented with 10 mM tyrosine.

### 2.5. Optimization of the Microbial Consortia Catalysis

**Figure 5:**
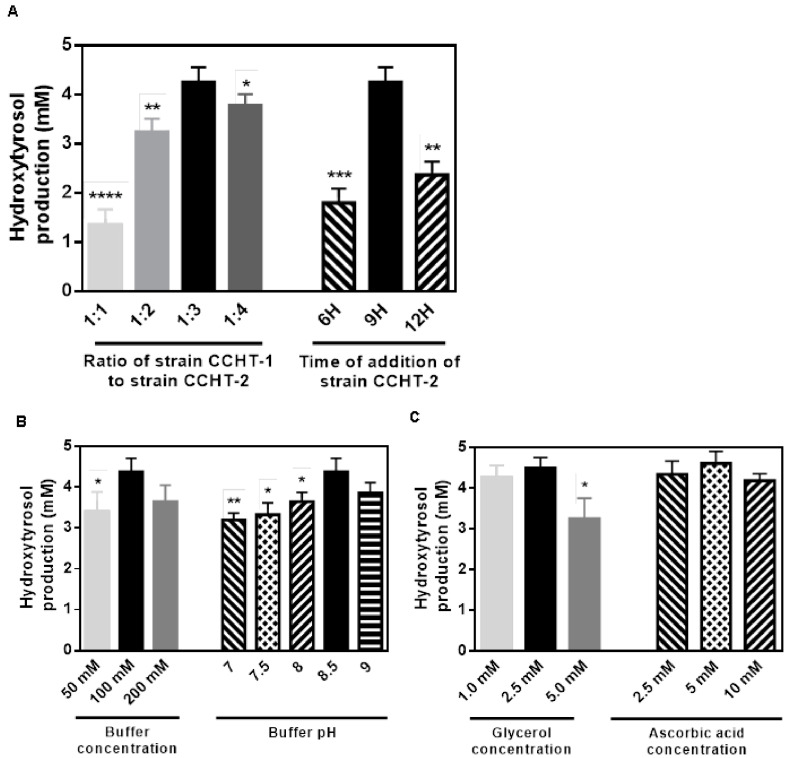
Optimization of the production of hydroxytyrosol from tyrosine. (**A**) Optimization of the ratio of two strains and the time of addition. (**B**) Optimization of buffer concentration and pH. (**C**) Optimization of the concentration of glycerol and ascorbic acid. 10 mM tyrosine was supplemented as substrate. Data are expressed as the mean ± SD (*n* = 3). * *p* < 0.05, ** *p* < 0.01, *** *p* < 0.001, and **** *p* < 0.0001, compared to each control.

In order to prevent coloration changes and optimize hydroxytyrosol conversion, a microbial consortia catalysis approach was utilized. Figure 5A demonstrated the ratio of CCHT-2 strain to CCHT-1 strain and the timing of the addition of the CCHT-2 strain was assessed to determine the optimal conditions for efficient conversion. Results indicated that a ratio of 3:1 (*v*:*v*) and an addition time of 9 h for the CCHT-2 strain were optimal, resulting in low tyrosine concentration and reduced promiscuous catalysis of HpaBC. To balance the activities of the various enzymes involved in the biosynthetic pathway, the concentration and pH of the buffer used in the microbial consortia were also optimized (Figure 5B). The highest yield of hydroxytyrosol (88%) was achieved at pH 8.5 using 100 mM Tris-HCl buffer, while higher or lower pH and buffer ion concentration were found to be less effective. Glycerol and ascorbic acid were added to the culture to enrich the carbon source, reduce force, and maintain cell metabolism resulting in yields of 90% and 92%, respectively (Figure 5C). The growth curves of separate strains and the consortia were presented in Figure S2.

## 3. Discussion

The article discusses the biosynthesis of hydroxytyrosol, a compound that has gained attention in recent years due to its potential health benefits [22,23,24,25,26]. Most of the biosynthetic pathways involve the production of an intermediate tyrosol, either from simple sugar or tyrosine, highlighting the importance of the final hydroxylation step of tyrosol to hydroxytyrosol. Two enzymes, tyrosinase and 4-hydroxyphenylacetate 3-hydroxylase, can catalyze this reaction. However, tyrosinase is not suitable for application in the biosynthesis of hydroxytyrosol from tyrosine, as it also catalyzes the oxidation of *o*-diphenols to *o*-quinones [27]. As for 4-hydroxyphenylacetate 3-hydroxylase, only HpaBC from *E. coli* BL21 (DE3) did not have aryl-dehydrogenase activity and, hence, was unable to oxidize hydroxytyrosol to 3,4-dihydroxyphenylacetic acid, making it a good candidate for hydroxytyrosol production [28]. Tyrosol produced by *S. cerevisiae* via the Ehrlich pathway from tyrosine has also been studied [29]. Cofactor self-sufficient whole-cell biocatalysts was also applied to produce 2-phenylethanol from L-phenylalanine in *E. coli* [30]. In the present study, we evaluated the efficiency of hydroxytyrosol production from tyrosine via the Ehrlich pathway and HpaBC catalysis. A heterogeneous hydroxytyrosl pathway was introduced into *E. coli*, and the efficiency of the pathway was optimized by substituting key enzymes such as the aminotransferase, the 4-hydroxyphenylpyruvate decarboxylases, and the reductase. Additionally, the glutamate dehydrogenase was introduced either to realize the coupling of two reactions catalyzed by the transaminase and the reductase, respectively, facilitating the regeneration of cofactor. By adding L-glutamate, α-ketoglutarate, and NADPH cofactor were regenerated simultaneously, improving the cofactor supply efficiently. 

However, HpaBC is a promiscuous enzyme with a broad substrate spectrum that includes tyrosine. When tyrosine was used as a substrate, the promiscuity of HpaBC can lead to the formation of L-dopa, which is unstable and readily oxidized. This can cause the culture color to turn black and generate reactive oxygen species, resulting in the loss of carbon sources. The promiscuous enzymatic activity of HpaBC has perplexed many researchers in relevant studies. Various methods have been adopted in previous studies to solve the problems caused by the promiscuity of HpaBC, including using *E. coli* K-12 derivatives without the *hpaB* gene to produce tyrosol from simple sugars [20], optimizing the expression of the enzymes in the pathway and adjusting the inoculation timing, or designing a microbial co-culture system with proper medium [31]. Semi-rational protein engineering has also been performed to modify the specificity of HpaBC [32]. 

**Figure 6:**
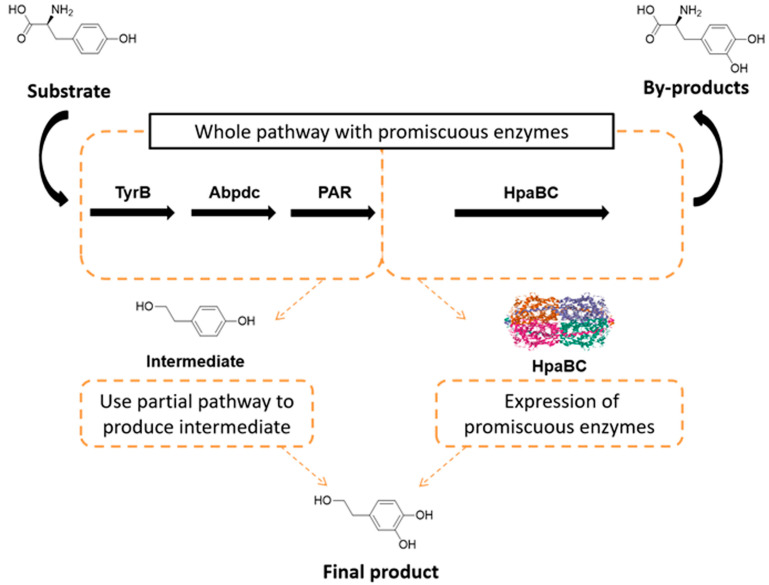
Diagram of the two-stage microbial consortia catalysis strategy.

In this study, the authors designed a novel microbial consortia catalysis strategy to address the issue of HpaBC promiscuity. Since the Ehrlich pathway is responsible for the production of tyrosol from tyrosine, and HpaBC is responsible for both the expected hydroxylation of tyrosol and the unexpected hydroxylation of tyrosine, it is crucial to improve the hydroxylation of tyrosol but inhibit the hydroxylation of tyrosine. We used a two-stage approach, where in the first stage, tyrosol was synthesized from tyrosine by strain CCHT-1 via the Ehrlich pathway composed of TyrB_EC_, Abpdc, Par, and GdhA, and another strain CCHT-2 was fermented for efficient expression of HpaBC. In the second stage, two cultures were mixed together to form an optimized microbial consortia catalysis, as depicted in Figure 6. This strategy allowed the oxidation of tyrosine by HpaBC to be avoided, without requiring challenging protein engineering methods. The authors suggest further studies are needed to evaluate the scalability and practicality of this strategy.

The efficient utilization of biosynthetic pathways poses a significant challenge as they are composed of more than two enzymes and require an optimal environment for their functioning [33]. It is difficult for a single host to achieve these conditions; therefore, microbial consortia systems have been employed to overcome this challenge. A stable consortium of two strains of the same species is preferred as it resolves the instability issue caused by multi-species consortia. However, modulating separate strains to achieve optimal functioning in the consortia is challenging when there is neither interdependence or specific sugar-selective characteristics of the two strains [34]. The novel two-stage microbial consortia catalysis strategy described in this study allows for a stepwise, space-time functioning of the segregated portion of the biosynthetic pathway, which provides a chance to modulate separate strains and achieve optimal conditions when combining the two strains. By optimizing the microbial consortia catalysis such as combining ratio, time, buffer pH and ion concentration, and cofactor supply, it is possible to avoid the promiscuity of pathway enzymes and screen and confirm the optimal conditions for key enzyme functioning. This results in the highest yield of the target product and the least side reaction. Each strain can be engineered independently, resulting in well-controlled metabolic flux, highly efficient substrate utilization, and final product production.

In summary, our two-stage strategy successfully overcame the promiscuity issue of the key enzyme in the biosynthetic pathway and optimized the bioconversion process of tyrosine to hydroxytyrosol by segregating the pathway into two parts. Compared to most co-culture strategies, which are hindered by population instability and inefficient control of the bioconversion conditions, our strategy simplified the optimization of the bioconversion process and circumvented the stabilization issue of different populations. This approach can also be extended to microbial consortia systems comprising two or more microbial species. Furthermore, this strategy can be enhanced by providing optimal conditions for each enzyme and/or precisely regulating the bioconversion process automatically. This will lead to the development of a super-efficient multi-stage and multi-species intelligent microbial consortia strategy for metabolic engineering and synthetic biology, which is a valuable supplement to co-culture strategies used in the microbial production of value-added substances.

## 4. Materials and Methods

### 4.1. General

DNA polymerase mix kits were purchased from Takara Bio, Inc. (Dalian, China) and YEASEN Biotechnology Co., Ltd. (Shanghai, China). Gibson assembly kit was purchased from GeneralBio Co., Ltd. (Anhui, China). Tyrosine, 4-hydroxyphenylpyruvate, 4-hydroxyphenylacetaldehyde, tyrosol, hydroxytyrosol, and L-dopa were all purchased from Sigma-Aldrich Co., Ltd. (St. Louis, MO, USA). *E. coli* BL21 (DE3) and W3110 were used for cloning and hydroxytyrosol biosynthesis, respectively. All *E. coli* strains were grown at 37 °C in yeast extract M9Y medium (M9 minimal salts (Becton, Dickinson and Company), 1% (*w*/*v*) glucose, 5 mM MgSO_4_, 0.1 mM CaCl_2_ supplemented with 0.025% (*w*/*v*) of yeast extract) [35]. Ampicillin (100 μg mL^−1^) and kanamycin (50 μg/mL) were used when necessary.

### 4.2. Strain and Plasmid Construction

All plasmids and primers used are listed in Supplementary Tables S1 and S2, respectively. *E. coli* W3110 with *feaB* gene deleted (W3110 △*feaB*), and plasmid pRSF and pFA were constructed as described in a previous study [35]. The *kan^+^* antibiotic resistance gene of plasmid pFA was replaced with *amp^+^*, resulting in the plasmid pFA1A.

(1) pRSF plasmids with different aminotransferases.

The *hpaBC* gene (Genbank accession No. CP020368.1) was amplified with primers *hpaB*-for/*hpaC*-rev and *E. coli* BL21 (DE3) genomic DNA. The *tyrB_EC_* gene (Genbank accession APC54183.1) was amplified with primers *tyrB_EC_*-for/*tyrB_EC_*-rev and *E. coli* W3110 genomic DNA. The *abpdc* gene from *A*. *brasilense* (Genbank accession No. AKE79068.1), the *par* gene from the rose (*Rosa* hybrid cultivar) (Genbank accession No. A0A0B6VQ48.1) and the *synKDC4* gene from *K*. *phaffii* GS115 (Genbank accession No. XP_002493734.1) were synthesized by GeneralBio (Anhui, China) after codon optimization (Supplementary Table S3). Then, the *abpdc* gene and *par* gene were amplified with primers *abpdc*-for/*abpdc*-rev and *par*-for/*par*-rev, respectively. The vector fragment was amplified with pRSF-for/pRSF-rev and plasmid pRSF. Then, the purified fragments of *hpaBC* gene, *tyrB_EC_* gene, *abpdc* gene, and *par* gene were assembled with the purified vector fragment, resulting in plasmid pRSF-*hpaBC*-*tyrB_EC_*-*abpdc*-*par* (pRSF-HT). The *aspC_EC_* gene (Genbank accession No. APC51199.1) was amplified with primers *aspC_EC_*-for/*aspC_EC_*-rev and *E. coli* W3110 genomic DNA. The *tyrB_PA_* gene (Genbank accession No. NP_251829) and the *phhC_PA_* gene (Genbank accession No. AAG04259.1) were amplified with primers *tyrB_PA_*-for/*tyrB_PA_*-rev and *phhC_PA_*-for/*phhC_PA_*-rev, respectively, with *P*. *aeruginosa* PAO1 genomic DNA. The *tyrB_PP_* gene (Genbank accession No. UZM96595.1) was amplified with primers *tyrB_PP_*-for/*tyrB_PP_*-rev and *P*. *putida* DOT-TIE genomic DNA. Vector fragment was amplified with primers pRSF-for/pRSF-rev and plasmid pRSF-HT. Then, the purified fragments of *aspC_EC_* gene, *tyrB_PA_* gene, *phhC_PA_* gene, and *tyrB_PP_* gene were assembled with the purified vector fragment, respectively, resulting in plasmids pRSF-*hpaBC*-*aspC_EC_*-*abpdc*-*par*, pRSF-*hpaBC*-*tyrB_PA_*-*abpdc*-*par*, pRSF-*hpaBC*-*phhC_PA_*-*abpdc*-*par*, and pRSF-*hpaBC*-*tyrB_PP_*-*abpdc*-*par*.

(2) pRSF plasmids with different 4-hydroxyphenylpyruvate decarboxylases

The *aro10* gene (Genbank accession No. NP_010668.3), the *aro8* gene (Genbank accession No. NP_011313.1), and the *adh1* gene (Genbank accession No. NP_014555.1) were amplified with primers *aro10*-for/*aro10*-rev, *aro8*-for/*aro8*-rev, and *adh1*-for/*adh1*-rev, respectively, with *S*. *cerevisiae* genomic DNA. The *synKDC4* gene was amplified with primers *synKDC4*-for/*synKDC4*-rev. Vector fragment was amplified with primers pRSF-for/pRSF-rev and plasmid pRSF-HT. Then, the purified fragments of *aro10* gene, *aro8* gene and *synKDC4* gene were assembled with the purified vector fragment, respectively, resulting in plasmids pRSF-*hpaBC*-*tyrB_EC_*-*aro10*-*par*, pRSF-*hpaBC*-*tyrB_EC_*-*aro8*-*par*, and pRSF-*hpaBC*-*tyrB_EC_*-*synKDC4*-*par*.

(3) pRSF plasmids with different reductases

The *yqhD* gene (Genbank accession No. APC53184.1), the *yjgB* gene (Genbank accession No. APC54393.1), the *dkgB* gene (Genbank accession No. APC50597.1), and *yahK* gene (Genbank accession No. BAE76108.1) were amplified with primers *yqhD*-for/*yqhD*-rev, *yjgB*-for/*yjgB*-rev, *dkgB*-for/*dkgB*-rev, and *yahK*-for/*yahK*-rev with *E. coli* W3110 genomic DNA. The *calA* gene (Genbank accession No. ACP17962.1) was amplified with primers *calA*-for/*calA*-rev with *P*. *nitroreducens* genomic DNA. Vector fragment was amplified with primers pRSF-for/pRSF-rev and plasmid pRSF-HT. Then, the purified fragments of *yqhD* gene, *yjgB* gene, *dkgB* gene, *yahK* gene, *calA* gene, and *adh1* gene were assembled with the purified vector fragment, respectively, resulting in plasmids pRSF-*hpaBC*-*tyrB_EC_*-*abpdc*-*yqhD*, pRSF-*hpaBC*-*tyrB_EC_*-*abpdc*-*yjgB*, pRSF-*hpaBC*-*tyrB_EC_*-*abpdc*-*dkgB*, pRSF-*hpaBC*-*tyrB_EC_*-*abpdc*-*yahK*, pRSF-*hpaBC*-*tyrB_EC_*-*abpdc*-*calA*, and pRSF-*hpaBC*-*tyrB_EC_*-*abpdc*-*adh1*.

(4) Plasmids for co-culture strains

The *gdhA* gene (Genbank accession No. APC52020.1) was amplified with primers *gdhA*-for/*gdhA*-rev and *E. coli* W3110 genomic DNA; the hpaBC gene was amplified with primers *hpaBC*-for-1/*hpaBC*-rev-1. Vector fragment was amplified with primers pRSF-for/pRSF-rev and plasmid pFA1A. Then, the purified fragments of *gdhA* gene and *hpaBC* gene were assembled with the purified vector fragment, respectively, resulting in plasmids pFA1A-*gdhA* and pFA1A-*hpaBC*. The *gdhA* gene was amplified with primers *gdhA*-for-1/*gdhA*-rev-1, vector fragment was amplified with primers pRSF-for/pRSF-rev and plasmid pRSF; then, the purified fragments of *gdhA* gene was assembled with the purified vector fragment, resulting in plasmid pRSF-*gdhA*-*tyrB_EC_*-*abpdc*-*par*.

(5) Plasmids for protein expression and purification

The *hpaBC* gene fragment was amplified with primers *hpaB*-for-1/*hpaC*-rev-1 and *E. coli* BL21 (DE3) genomic DNA. Vector fragment was amplified with primers vector-for/vector-rev and plasmid pET28a. Then, the purified fragment of *hpaBC* gene was assembled with the purified vector fragment, resulting in plasmid pET28a-*hpaBC*.

### 4.3. High-Performance Liquid Chromatography (HPLC) Quantification

A colony of *E. coli* W3110 △*feaB* harboring plasmid was grown in M9Y medium at 37 °C, then was induced with 1 mM L-arabinose when OD_600_ = 0.6, then 10 mM tyrosine was supplemented. After grown for 24 h, the culture was centrifuged at 10,000× *g* for 10 min. Then, the supernatant was collected and filtrated through a 0.22 μm filter membrane. Concentrations of tyrosine, tyrosol, and hydroxytyrosol were determined by HPLC. Shimadzu LC-20A system equipped with SPD-M20A photo-diode array (PDA) detector (280 nm) and InertsilODS-SPC18 column (250 mm × 4.6 mm × 5 µm) working at 30 °C. The mobile phase A and B was 5% acetic acid and acetonitrile, respectively. Mobile phase B gradient: 15–40% (0–20 min), 40–100% (20–25 min). The flow rate is 0.8 mL/min, and the injection volume is 10 μL. 

### 4.4. Protein Expression and Purification

A single colony of *E. coli* BL21 (DE3) harboring plasmid pET28a-*hpaBC* was cultured and induced as described previously [32]. Then, cells were collected by centrifuge at 4000× *g* for 10 min at 4 °C, and resuspended in lysis buffer (100 mM Tris-HCl, 250 mM NaCl, 10 mM imidazole, pH 8.0) and disrupted by sonication for 30 min with a JY92-IIN Ultra Sonic Cell Crusher (Ningbo, China). After centrifugation at 10,000× *g* for 10 min, the supernatant was loaded on a Ni-NTA column, the HpaBC protein was purified, and the concentration was assayed as described previously [32].

### 4.5. Activity Assay

After purification, the activity of HpaBC was assayed as described previously with minor modification [32]. The substrates used in the assay were 4-HPA, tyrosine, 4-hydroxyphenylpyruvate, 4-hydroxyphenylacetaldehyde, tyrosol, hydroxytyrosol, and L-dopa. One unit of HpaBC activity was defined as the same as previously described [32].

### 4.6. Co-Culture Conditions

A colony of *E. coli* W3110 △*feaB* harboring plasmid pRSF-*gdhA*-*tyrB_EC_*-*abpdc*-*par* (strain CCHT-1) was grown in M9Y medium at 37 °C, and 1 mM L-arabinose and 10 mM tyrosine were supplemented when OD_600_ = 0.6. Meanwhile, a colony of *E. coli* W3110 △*feaB* harboring plasmid pFA1A-*hpaBC* (strain CCHT-2) was grown in M9Y medium at 37 °C, and 1 mM L-arabinose was supplemented when OD_600_ = 0.6. After induced for 9 h, cells of strain CCHT-2 were collected by centrifuge at 4000× *g* for 10 min. Then, the cell pellet was added into the culture of strain CCHT-1 with a different ratio. The co-culture condition was optimized to maintain the metabolism strains at basic level and to facilitate the biotransformation to the product hydroxytyrosol. The concentrated microbial consortia (OD_600_ = 20) were inoculated in 300 mL shake flasks, ran for 6–9 h at 100 rpm, and 30 °C of a 100 mM Tris-HCl buffer (pH 8.5) with 2.5 mM glycerol, 5 mM each of ascorbic acid, L-glutamate, nicotinic acid, and nicotinamide. The yield of hydroxytyrosol was determined after co-culture for a certain time.

### 4.7. Statistical Analysis

Each experiment was repeated at least thrice. All statistical analyses were performed by one-way analysis of variance (ANOVA) using GraphPad prime 9.0. Numerical data were expressed as mean ± SD, and *p*-values < 0.05 were considered statistically significant.

## Data Availability

Data are available upon request to corresponding authors.

## Supplementary Materials

The following supporting information can be downloaded at: https://www.mdpi.com/article/10.3390/ijms24086944/s1.
