# Supplementary material for: A Novel Microbial Consortia Catalysis Strategy for the Production of Hydroxytyrosol from Tyrosine
Source: Int J Mol Sci. 2023 Apr 8;24(8):6944. doi: 10.3390/ijms24086944 (PMC10139182; doi:10.3390/ijms24086944)
Supplement: Supplementary file 1 [file ijms-24-06944-s001.zip › ijms-2291891-supplementary.pdf]

Supplementary Material

A Novel Microbial Consortia Catalysis Strategy for the Production of Hydroxytyrosol from Tyrosine

Pengfei Gong, Jiali Tang, Jiaying Wang, Chengtao Wang * and Wei Chen *

Key Laboratory of Geriatric Nutrition and Health, Ministry of Education, Beijing Advanced Innovation Center for Food Nutrition and Human Health, Beijing Engineering and Technology Research Center of Food Additives, School of Food and Health, Beijing Technology and Business University, Beijing 100048, China

* Correspondence: wangchengtao@th.btbu.edu.cn (C.W.); weichen@btbu.edu.cn (W.C.)

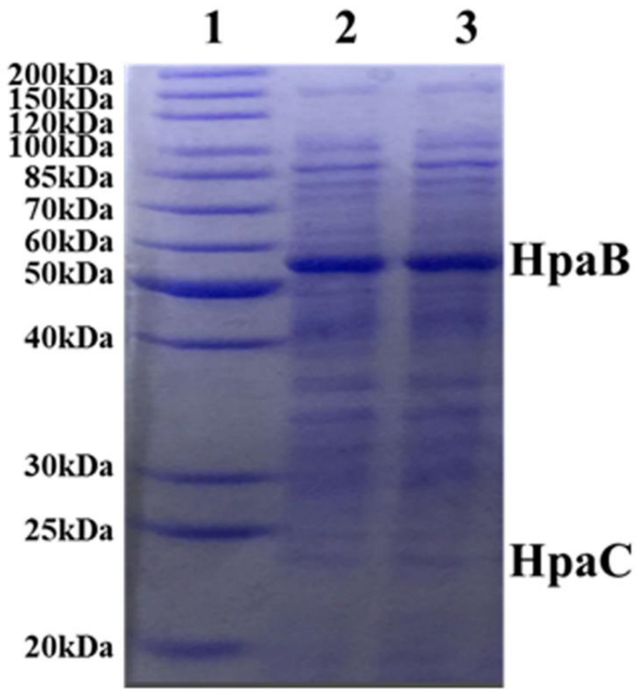

Figure S1. Protein expression of HpaBC. Lane 1 shows the Protein Marker. Lane 2 and 3 show the cell lysate of strain BL21(DE3) harboring plasmid pET28a-*hpaBC*, after culture and induction.

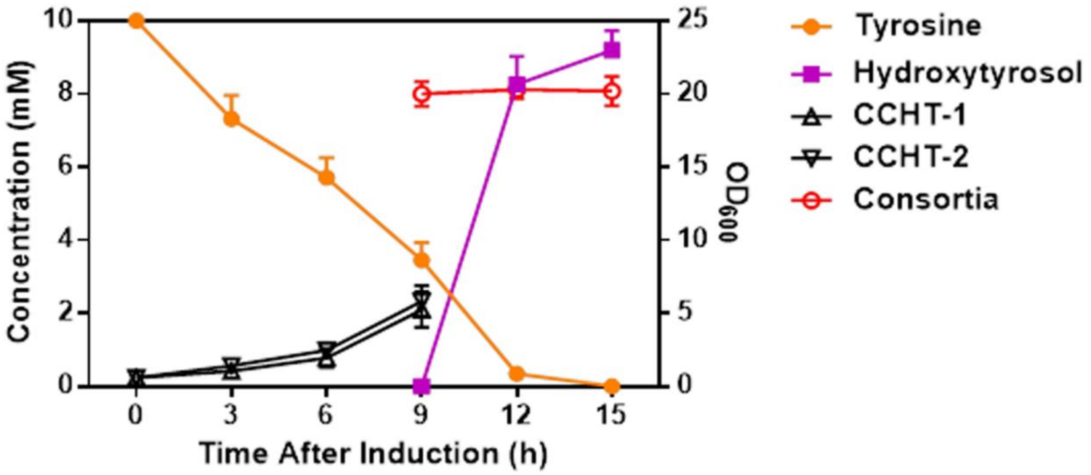

Figure S2. Growth curves of strains. The time-course of separate strains and the consortia. The orange closed circle represents the time course of tyrosine concentration, the purple square represents the time course of hydroxytyrosol concentration, the hollow black equilateral triangl, inverted triangle, and red cycle represents the time course of the OD_{600} of the CCHT-1, CCHT-2 and consortia respectively. Data are expressed as the mean ± SD (n = 3).

Table S1 Plasmids used in this study.

Plasmids	Description	source
pFA1A	Plasmid with p15A replication origin, *amp*$^+$	[35]
pRSF	Plasmid with pRSF3010 replication origin, *kan*$^+$	[35]
pRSF-*hpaBC-tyrB*$_{EC}$*-abpdc-par*	Pathway constructed in pRSF	This study
pRSF-*hpaBC-aspC*$_{EC}$*-abpdc-par*	Pathway constructed in pRSF	This study
pRSF-*hpaBC-tyrB*$_{PA}$*-abpdc-par*	Pathway constructed in pRSF	This study
pRSF-*hpaBC-phhC*$_{PA}$*-abpdc-par*	Pathway constructed in pRSF	This study
pRSF-*hpaBC-tyrB*$_{PP}$*-abpdc-par*	Pathway constructed in pRSF	This study
pRSF-*hpaBC-tyrB*$_{EC}$*-aro10-par*	Pathway constructed in pRSF	This study
pRSF-*hpaBC-tyrB*$_{EC}$*-aro8-par*	Pathway constructed in pRSF	This study
pRSF-*hpaBC-tyrB*$_{EC}$*-synKDC4-par*	Pathway constructed in pRSF	This study
pRSF-*hpaBC-tyrB*$_{EC}$*-abpdc-yqhD*	Pathway constructed in pRSF	This study
pRSF-*hpaBC-tyrB*$_{EC}$*-abpdc-yjgB*	Pathway constructed in pRSF	This study
pRSF-*hpaBC-tyrB*$_{EC}$*-abpdc-dkgB*	Pathway constructed in pRSF	This study
pRSF-*hpaBC-tyrB*$_{EC}$*-abpdc-yahK*	Pathway constructed in pRSF	This study
pRSF-*hpaBC-tyrB*$_{EC}$*-abpdc-calA*	Pathway constructed in pRSF	This study
pRSF-*hpaBC-tyrB*$_{EC}$*-abpdc-adh1*	Pathway constructed in pRSF	This study
pRSF-*gdhA-tyrB*$_{EC}$*-abpdc-par*	Co-culture plasmid	This study
pFA1A-*gdhA*	Co-culture plasmid	This study
pFA1A-*hpaBC*	Co-culture plasmid	This study
pET28a-*hpaBC*	HpaBC expressed from pET28a vector	This study

Table S2 Primers used in this study.

Primers	Sequence (5'-3')
hpaB-for	GCTAACAGGAGGAATTACATATGAAACCAGAAGATTTCCG
hpaC-rev	TGAAACATATTGTTTCTCCTTTAAATCGCAGCTTCCATTT
tyrB$_{EC}$-for	CGATTTAAAGGAGAAACAATATGTTTCAAAAAGTTGACGC
tyrB$_{EC}$-rev	TTCGCCATATTGTTTCTCCTTTACATCACCGCAGCAAACG
abpdc-for	TGATGTAAAGGAGAAACAATATGGCGAAACTGGCGGAAGC
abpdc-rev	TTGCTCATATTGTTTCTCCTTTATTCGCGAGGTGCCGCAT
par-for	GCGAATAAAGGAGAAACAATATGAGCAATAAAGTGGTGTG
par-rev	CGGAAATCTTCTGGTTTCATTTAAAAGCTCACAATGCTTT
pRSF-for	AGATCTGGTACTAGTGGTGA
pRSF-rev	CGGAAATCTTCTGGTTTCAT
aspC$_{EC}$-for	CGATTTAAAGGAGAAACAATATGTTTGAGAACATTACCGC
aspC$_{EC}$-rev	TTCGCCATATTGTTTCTCCTTTACAGCACTGCCACAATCG
tyrB$_{PA}$-for	CGATTTAAAGGAGAAACAATATGAGTCTGTTTTCTGCCGT
tyrB$_{PA}$-rev	TTCGCCATATTGTTTCTCCTCTACAGGACCTGGACGATGG
phhC$_{PA}$-for	CGATTTAAAGGAGAAACAATATGAGTCATTTCGCCAAGGT
phhC$_{PA}$-rev	TTCGCCATATTGTTTCTCCTTCAGTCCGCGCAGACCTGGG
tyrB$_{PP}$-for	CGATTTAAAGGAGAAACAATATGTTCAAACATGTCGATGC
tyrB$_{PP}$-rev	TTCGCCATATTGTTTCTCCTTTACTTCTGAACGGCAGCGA
aro10-for	TGATGTAAAGGAGAAACAATATGGCACCTGTTACAATTGA
aro10-rev	TTGCTCATATTGTTTCTCCTCTATTTTTTATTTCTTTTAA
aro8-for	TGATGTAAAGGAGAAACAATATGACTTTACCTGAATCAAA
aro8-rev	TTGCTCATATTGTTTCTCCTCTATTTGGAAATACCAAATT
adh1-for	TGATGTAAAGGAGAAACAATATGTCTATCCCAGAAACTCA
adh1-rev	TTGCTCATATTGTTTCTCCTTTATTTAGAAGTGTCAACAA
synKDC4-for	TGATGTAAAGGAGAAACAATATGGCGCCGGTGAAACAGGA

synKDC4-rev	TTGCTCATATTGTTTCTCCTTTAATGAATGCTTTTACTCG
yqhD-for	GCGAATAAAGGAGAAACAATATGAACAACTTTAATCTGCA
yqhD-rev	CGGAAATCTTCTGGTTTCATTTAGCGGGCGGCTTCGTATA
yjgB-for	GCGAATAAAGGAGAAACAATATGTCGATGATAAAAAGCTA
yjgB-rev	CGGAAATCTTCTGGTTTCATTCAAAAATCGGCTTTCAACA
dkgB-for	GCGAATAAAGGAGAAACAATATGGCTATCCCTGCATTTGG
dkgB-rev	CGGAAATCTTCTGGTTTCATTTAATCCCATTCAGGAGCCA
yahK-for	GCGAATAAAGGAGAAACAATATGAAGATCAAAGCTGTTGG
yahK-rev	CGGAAATCTTCTGGTTTCATTCAGTCTGTTAGTGTGCGAT
calA-for	GCGAATAAAGGAGAAACAATATGCAGCTGACCAACAAAAA
calA-rev	CGGAAATCTTCTGGTTTCATTTACACGTAGGTGCTGGCCA
gdhA-for	GCTAACAGGAGGAATTACATATGGATCAGACATATTCTCT
gdhA-rev	TGAAACATATTGTTTCTCCTTTAAATCACACCCTGCGCCA
hpaBC-for-1	GCTAACAGGAGGAATTACATATGAAACCAGAAGATTTCCG
hpaBC-rev-1	CGGAAATCTTCTGGTTTCATTTAAATCGCAGCTTCCATTT
gdhA-for-1	GCTAACAGGAGGAATTACATATGGATCAGACATATTCTCT
gdhA-rev-1	CGGAAATCTTCTGGTTTCATTTAAATCACACCCTGCGCCA
hpaB-for-1	TGGTGGTGGTGGTGCTCGAGTTAAATCGCAGCTTCCATTT
hpaC-rev-1	TGGTGCCGCGCGGCAGCCATATGAAACCAGAAGATTTCCG
vector-for	CGGAAATCTTCTGGTTTCATATGGCTGCCGCGCGGCACCA
vector-rev	AAATGGAAGCTGCGATTTAACTCGAGCACCACCACCACCA

Table S3 Sequence of genes after codon optimization.

Gene name	DNA sequence after codon optimization
abpdc	ATGGCGAAACTGGCGGAAGCCCTGCTGCGCGCCCTGAAAGATCGTG<br>GCGCGCAGGCCATGTTCGGCATCCCGGGCGATTTCGCGCTGCCTTTT<br>TTTAAAGTGGCGGAAGAAACCCAGATTCTGCCGCTGCACACCCTGAG<br>CCACGAACCGGCCGTGGGCTTCGCCGCCGATGCGGCCGCGCGCTA<br>CTCGGCGACCCTGGGTGTTGCGGCGGTAACGTACGGAGCCGGTGCG<br>TTTAATATGGTCAATGCCGTTGCGGGTGCGTACGCGGAAAAAAGCCC<br>GGTGGTTGTTATTAGTGGTGCGCCGGGTACCACCGAAGGCAACGCG<br>GGTCTGCTGCTGCACCATCAGGGCCGCACCCTGGATACCCAGTTCCA<br>GGTTTTCAAAGAAATCACCGTGGCGCAGGCCCGCCTGGATGATCCGG<br>CAAAAGCGCCGGCCGAGATCGCCCGCGTGCTGGGCGCCGCGCGCG<br>CGTTAAGCCGCCCGGTGTATCTGGAAATTCCGCGTAACATGGTGAATG<br>CCGAAGTGGAACCGGTGGGCGATGATCCGGCATGGCCGGTGGACCG<br>TGATGCACTGGCGGCCTGCGCCGACGAAGTGTTAGCAGCAATGCGC<br>GCCGCGACCTCACCTGTTCTGATGGTGTGCGTTGAAGTGCGTCGTTA<br>TGGTCTGGAAGCGAAAGTGGCGGAACTGGCGCAGCGTCTGGGCGT<br>GCCGGTGGTTACCACCTTCATGGGCCGCGGGCTGCTGGCAGATGCG<br>CCGACCCCGCCGCTGGGCACCTATATTGGCGTGGCCGGCGATGCGG<br>AAATTACCCGTCTGGTTGAAGAAAGCGATGGTCTGTTTCTGCTGGGT<br>GCAATTCTGTCAGATACGAATTTTGCGGTTAGCCAGCGTAAAATTGATT<br>TACGTAAAACCATTCATGCGTTTGATCGTGCGGTGACCCTGGGCTATC<br>ATACCTATGCTGATATTCCGCTGGATGGTCTGGTGGATGCCCTGTTGG<br>AACGCCTGCCGCCGAGCGACCGTACCACCCGTGGCAAAGAACCGCA<br>TGCGTATCCGACTGGCCTGCAAGCGGATGGCGAACCGATTGCCCCGA<br>TGGATATTGCGCGCGCGGTCAATGATCGTGTGCGTGCGGGCCAGGA<br>ACCGCTGCTGATTGCCGCAGACATGGGCGATTGCCTGTTTACCGCGA<br>TGGATATGATTGATGCCGGTCTGATGGCACCGGGCTATTATGCGGGCA<br>TGGGCTTTGGCGTTCCGGCCGGTATTGGCGCGCAGTGCGTGAGCGG<br>CGGCAAGCGCATTCTGACCGTGGTTGGCGATGGCGCATTTCAGATGA<br>CTGGCTGGGAGCTGGGTAACTGTCGTCGTCTGGGCATTGATCCGATT<br>GTCATTCTGTTCAACAATGCCAGTTGGGAAATGCTGCGTACCTTTCAG<br>CCGGAATCTGCCTTTAATGACCTGGACGATTGGCGTTTTGCCGATATG<br>GCGGCGGGTATGGGCGGTGATGGCGTGCGCGTGCGCACCCGTGCC<br>GAACTGAAAGCGGCGCTGGATAAAGCGTTTGCAACCCGCGGCCGCT<br>TTCAGTTGATTGAAGCAATGATTCCGCGCGGCGTACTGAGCGATACTC<br>TGGCGCGCTTTGTTCAGGGCCAGAAACGCCTGCATGCGGCACCTCG<br>CGAATAA
par	ATGAGCAATAAAGTGGTGTGCGTGACCGGCGCCAGCGGCTACATTGC<br>GAGCTGGCTGGTGAAACTGCTGCTTCAGCGCGGCTATACCGTGAAAG<br>CGAGCGTGCGCAATCCGAACGATCCGACCAAAACCGAACATCTGCTG

GCCCTGGATGGCGCGAAAGAACGCCTGCAGCTGTTTAAAGCCGATCT
GCTGGAAGAAGGCAGCTTTGATAGCGCCGTGGAAGGTTGCGAAGGC
GTGTTTCATACCGCGAGCCCGTTTTATCATGATGTGACCGATCCGAAA
GCCGAACTGCTGGACCCGGCAGTGAAAGGCACCCTGAACGTGCTGA
ACAGCTGTAGCAAAAGCCCGAGCATTAAACGCGTGGTGCTGACCAGC
AGCATTGCGGCGGTGGCGTACAATGGCAAACCGCGCACCCCGGATG
TGGTGGTTGATGAGACCTGGTTTACCGATCCGGATGTGTGCAAAGAA
AGCAAACTGTGGTATGTGCTGAGCAAAACCCTGGCCGAAGATGCGGC
CTGGAAATTCGTGAAAGAAAAAGGCATTGATATGGTAACCATTAATCCG
GCGATGGTGATTGGCCCGCTGCTGCAGCCGACGCTGAACACCAGTG
CCGCCGCAATTCTGAACATTATTAAAGGCGCGCGCACGTACCCGAAT
GCCAGCTTTGGCTGGATTAATGTGAAAGATGTTGCAAATGCGCATGTC
CAGGCCTTCGAAATTCCGTCAGCAAGCGGCCGCTACTGCCTGGTGG
AACGTGTGGCCCATTTTACCGAAGTGCTGCAGATTATTCACGAACTGT
ATCCGGATCTGCAACTGCCGGAAAAATGCTCAGATGATAAACCGTTTG
TACCGACCTATCAGGTGAGCAAAGAAAAAGCCAAAAGCCTGGGCATT
GAATTTATTCCGCTGGATATTAGCCTGAAAGAAACCATTGAGAGCCTG
AAAGAAAAAAGCATTGTGAGCTTTTAA

synKDC4

ATGGCGCCGGTGAAACAGGATTTTAACATCGACGTGCAGACGATCGA
AAATACCGACATTAGCCTGTCGGAATATATTTACCTGCGCATTGCCCAG
CTGGGTGTGAAAAGCATTTTCGGCGTACCGGGCGATTTTAATCTGAAT
CTGGTGGATGAACTGGATAAAGTGCCTCAGCTGAAATGGATTGGCTG
CTGCAATGAACTGAACGCGACCTATGCGGCCGATGGCTATGCGAAAG
CGAGCGGCACCATTGGCGTGGTGGTAACCACCTACGGCGTGGGCGA
ACTGAGCGCGATTAATGGCATTGCCGGCGCGTTCGCGGAATACGCGC
CGGTTCTGCACATTGTGGGCACCAGCGCGATGGCGACCAAACGTCT
GGAACATGTTCACAACATTCACCACCTGGCGGGCAGCAAAAACTTCC
TGGATCGCCCGGATCACTATATTTATGAAAAAATGGTGGATGACATTTG
TATTGTGAAAGAGTCTCTGTCGGAAATTGAAAATGCGTGTGGTCAGAT
TGATAACGCGATTGTGCAGACCTACCTGCTGAGCCGTCCGGGCTATC
TGTTCCTGCCGCGCAACATGGCCACCATGAAAGTGCCGCGTGAACG
CCTGTTTAACCAGCCGCTGGCCCTGGAACGCGTGGATCTGCATCCGG
GCGAAACCCTGCAGGTGGTCGAAAAAATTCTGGAAAAATTTTATCATG
CGAAAGAACCGGCCCTGATTGTGGATTACCTGACCCGCCCGTTTCGC
ATGATGGAAAACTGTAGCAAACTGATTGGCGCCCTGGAAAATAAAGTG
AATATTTTCAGCCGTCCGATGAGCAAAGGCTTTGTGGATGAAAGCCAT
CCGCGCTATATTGGCTGCTACATTGGCAAACAGAGCAAACATCCGAGC
ACCAGCGATATTCTGGAAAAAAAAAGCGACTTTATTCTGAGCGTGGGC
ACCTTTGATGTTGAAACCAATAACGGCGGCTTTACCAGCAAACTGCCG
CAGGAGCATCTGGTGGAACTGAACCCTCATTTTACCCGTGTTGGCAC
CCAGTGTTTTAGCAATGTTAATATGTGCCATGTCCTGCCGCTGCTGGC
GAGCAAACTGCGTGGCGATCTGATTAGCATGGCCACTGTGCATCCGA
ACGATTTTAGCCTGCGCAAAAAAGAAAAAGCGCAGGATAAAATGAAAG

CGCTGAACCAGAGCCATCTGGTTAAAAGCACCGAACTGCTGCTGAAT
GCGAATGATACCCTGATTGTGGAAACCTGTAGCTTTATGTTCGCGGTG
CCGGATATTGCGTTTCCGAATAATACCCAGTTTATTAGCCAGAGCTTTT
ATAATAGCATTGGCTACGCGCTGCCGGCCACCCTGGGCGTGAGCATT
GCCAAACGCGATTTTCGTAAACCGGGCAAAGTGGTTCTGATTCAGGG
CGATGGCAGCGCGCAGATGACCATTCAGGAACTGGCGACCATGGTAC
GCCAGAAAGTGAAACCGACCATTCTGCTGCTGAACAATGAAGGCTAC
ACCGTGGAACGCATGATCCTGGGTCCGACCAAAGAATATAATGATATT
GCCCCGAACTGGGATTGGACCGGCATGCTGCGTGCCTTTGGCGATAT
TCGTGGTCATAGCAAATCCATTAGCATCGATACCTGCGGCCGCCTGGA
TAAGCTGGTACAGACGCGCGAATTCCAGGAACCGACCCATCTGAATTT
TGTTGAACTGATTCTGGGCCGCATGGATGCCCCGGAACGCTTCGCGA
ATATGGTTAAAGAGATCGCGAATCTGGAACACGCGAGTAAAAGCATTC
ATTAA
